# Decomposing a Chunk into Its Elements and Reorganizing Them As a New Chunk: The Two Different Sub-processes Underlying Insightful Chunk Decomposition

**DOI:** 10.3389/fpsyg.2017.02001

**Published:** 2017-11-14

**Authors:** Xiaofei Wu, Mei He, Yinglu Zhou, Jing Xiao, Jing Luo

**Affiliations:** ^1^Beijing Key Laboratory of Learning and Cognition, College of Psychology, Capital Normal University, Beijing, China; ^2^Department of Psychology, The Demonstration Center of Experimental Teaching of Psychological and Cognitive Behavior in Shanxi Province, School of Educational Science, Shanxi Normal University, Linfen, China; ^3^School of Labor and Human Resources, Renmin University of China, Beijing, China; ^4^Statistics Department, Art and Science College, Miami University, Oxford, OH, United States

**Keywords:** creative insight, chunk decomposition, chunk restructuring, R-process, D-process

## Abstract

Familiar chunks can be processed highly efficiently, and this automatic process can prohibit the problem solver from developing novel and original ways to creatively solve difficult problems. For this reason, the role of the reverse process, chunk decomposition (CD), the process by which familiar patterns are broken down into their component elements in order to be regrouped in another meaningful manner, has been generally recognized as part of the creative process. However, previous studies on this issue have mainly focused on the decomposition process of CD (the D-process), while the reorganization process of CD has been greatly neglected or has not been distinctively identified in previous work. In this paper, we argue that the R-process could be equally as important as the D-process for CD. Even if a problem solver manages to decompose a familiar chunk into its elements, he or she still may not solve the problem if these elements are not successfully organized in a new and meaningful manner. To investigate whether the cognitive mechanism of the R-process is different from that of the D-process, we designed an experiment for detecting the effects of chunk tightness, which is regarded as the key factor in CD and which can be experimentally manipulated by the radical-level (loose) and stroke-level (tight) Chinese character CD tasks in the D-process, the R-process, and the more purified organization task (the O-process task) that does not involve the decomposition process. Our results showed that the stroke-level (tight) task was more difficult than the radical-level (loose) task for the D-process. However, for the R-process, the stroke- and radical-level tasks showed no differences in performance. Moreover, for the more purified reorganization task, the O-process task, the radical-level organization and reorganization could be even more difficult than the stroke-level organization and reorganization. This result demonstrated that the cognitive processes underlying chunk decomposition and reorganization are fundamentally different. Therefore, more general concepts such as chunk restructuring that could include both D- and R-processes might be more suitable in accounting for this type of creative insight.

## Introduction

Chunk plays important role in many cognitive processes including perception, learning, and problem solving in humans and animals. Gobet et al. (2001) defined chunk as “a collection of elements having strong associations with one another, but weak associations with elements within other chunks.” Simultaneously, the information encoded as chunk would be stored in long-term memory and if necessary, it would be rapidly transferred from long-term memory to working memory (Guida et al., 2002; Gobet et al., 2016). Usually, the information processing based on chunks are highly automatic and are able to greatly promote cognitive efficiency or performance. However, the side-effects of chunk is that it could also result in inappropriate constrains on people's thinking especially the creative ones. Under this circumstance, the reverse process, chunk decomposition, is needed. Chunk decomposition (CD) refers to the decomposition of familiar and automatic patterns into their component elements so that they can be regrouped in another meaningful manner. Such regrouping is necessary because during problem encoding, problem elements become automatically grouped into familiar patterns, and these grouping processes may prevent the thinker from forming appropriate mental representations that are critical for successful insight problem solving. The aim of the present study was to reconsider the concept of CD and the theory behind it.

CD is one of the two basic approaches toward an insightful breakthrough (another approach is constraint relaxation that occurs when unsuitable constraints on the goal state of the problem are removed, Knoblich et al., 1999, 2001), according to representational change theory of creative insight (Ohlsson, 1984a,b, 1992) that regarded insight as a process of representational change through which the problem solver removes the unnecessary constraints he/she has inappropriately imposed on the problem and becomes aware of a novel conception that is suitable to represent and solve the problem. The most typical example of CD is the matchstick arithmetic task that was developed by Knoblich et al. (1999, 2001). In these tasks, participants were given a false arithmetic statement (such as **VI** = **VII** + **I**) written using Roman numerals (e.g., **I**, **II**, and **IV**), operations (+ and –) and an equal sign (=) and were required to transform the statement into a true equation by moving only one stick from one position to another within the pattern. For example, to the equation **VI** = **VII** + **I**, the solution is to decompose the **VII** into **VI** and **I**, and then move the I to the left side of the equal sign to form the true equation **VII** = **VI** + **I**. Compared with this example, Knoblich et al. (1999, 2001) found it was much more difficult to transform the equation “**XI** = **III** + **III**” to “**VI** = **III** + **III**.” Knoblich et al. (1999, 2001) interpreted this result from the CD point of view: **VII** is a relatively loose chunk that is composed of meaningful small chunks (i.e., **V** and **I**) and it is easy to decompose **VII** into **VI** and **I**. In contrast, to reform **XI** to **VI** is difficult because only when the **X** is decomposed into its meaningless components (i.e., \ and /) can a **V** be regrouped.

Although matchstick arithmetic task provided behavioral evidence that chunk decomposition is a source of difficulty in insight problem solving (Knoblich et al., 1999), the task domain does not provide large enough variety of problems and are especially not appropriate for neuroimaging studies (Luo et al., 2006; Luo and Knoblich, 2007). Therefore, follow-up studies on CD used Chinese characters as materials. Chinese characters are ideal examples of perceptual chunks that are composed of radicals, which, in turn, are composed of strokes. Strokes are the most simple and basic components of a Chinese character. Usually, isolated strokes do not carry meaning. In contrast, radicals convey information about the meaning and pronunciation of the character. Radicals usually consist of several strokes and can be regarded as the most basic chunk in the Chinese writing system. According to the theory of CD (Knoblich et al., 1999), it is much easier to separate a character into its radicals than to separate a character into its strokes because particular strokes are tightly embedded within a given perceptual chunk. In other words, the decomposition of characters into strokes requires a specific creative insight process that breaks the tight bond among strokes created by the perceptual chunk. This property enabled researchers to study the cognitive brain process of CD by contrasting the radical-level (loose) and stroke-level (tight) CD of Chinese characters (Luo et al., 2006; Tang et al., 2009, 2016; Wu et al., 2009, 2010, 2013; Huang et al., 2015). These neuroimaging studies found the negative activation (inhibition) of primary visual cortex and positive activation of higher visual cortex during the moment of CD (Luo et al., 2006; Wu et al., 2009), the involvement of the dorsal (“where”) and ventral (“what”) visual pathway in CD (Wu et al., 2010), the role of right hemisphere in memorizing the unsolved questions and identifying the potential hints for CD problems (Tang et al., 2009), the cognitive neuroscience mechanism for the “nonlinear” way of insightful breakthrough in CD (i.e., to overcome two multiple difficulties in one single thinking step) (Wu et al., 2013), task difficulty effects of CD, which was manipulated by a parametric experimental design, on the cognitive control areas such as prefrontal cortex (Tang et al., 2016), as well as the separable cognitive-neurological-brain basis for processing the “novel” and “appropriate” features, which are the two most fundamental features of creative thinking, in CD (Huang et al., 2015).

Principally, the process of chunk decomposition - the process of breaking or decomposing familiar patterns/chunks into their component elements - is critical for successful problem solving. Further, the success or not of CD was influenced by the chunk-tightness which meant that the CD could be achieved either in a “loose” or a “tight” manner. Principally, if a given chunk were broken into small chunks or sub-chunks (e.g., to decompose **VII** into **VI** and **I**), then the tightness of decomposition would be relatively low and easy to accomplish. However, if a given chunk was to be broken into more fundamental components or elements (e.g., to decompose **X** into \ and /), then the tightness of decomposition would instead be relatively high and difficult to achieve, such that this type of decomposition is typically associated with creative insights.

In this paper, we argue that the theory of chunk-decomposition might be incomplete. This condition could be demonstrated if we carefully inspect the classic example of CD using matchstick arithmetic tasks. In the most difficult and insightful matchsticks arithmetic task (i.e., to transform “**XI** = **III** + **III**” to “**VI** = **III** + **III**”), the key step in thinking is to transfer the **X** to the **V**. This transformation, of course, involves the process of decomposing the **X** into its meaningless components, i.e., the \ and /. However, it also involves the process of reorganizing the \ and / as a new number, **V**. In fact, even if one could successfully decompose the **X** into two isolated matchsticks, this did not guarantee the solving of the problem given that these two matchsticks could also be reorganized in many different ways. To form the **V** is the only efficient way to solve the problem at hand. Therefore, it is the process of rebuilding the problem components or elements in a different way that can specifically help to solve this problem; reorganization is thus essential for achieving an insightful resolution.

The above mentioned discussion implies the CD can at least contain two aspects or processes: the decomposition aspect or process (the D-process) and the reorganization aspect or process (the R-process). However, previous studies on this issue have mainly focused on the D-process. The R-process has been greatly neglected or at least has not been distinctively identified. In this study, we therefore designed three types of CD tasks: one for investigating the decomposition process (D-process tasks) of CD and two for investigating the reorganization process, with one task assessing both the decomposition process and the reorganization process (R-process tasks) and the other task assessing only the organization process (O-process tasks). We wanted to investigate the differences from behavior data between D- and R-process and reconsider the concept and theory of chunk decomposition.

## Method

### Participants

Sixty college students (27 males, mean age 23.4 years) were paid and randomly assigned to one of the two parts of the experiment. All participants possessed normal or corrected-to-normal vision, were right-handed, and had no history of neurological or psychiatric illness reported. Before the experiment, all participants signed an informed consent form approved by Capital Normal University's Committee on Activities Involving Human Subjects. Because the same Chinese characters were utilized as materials in the R-process tasks and the O-process tasks, the participants could not take part in the both tasks simultaneously. Half of the participants could take part in the D-process tasks and R-process tasks simultaneously (part 1), and the other half of the participants could take part in the O-process tasks (part 2). Nine of the participants were excluded from the analyses because they did not understand or follow the instructions. Therefore, the final sample consisted of 25 (12 males) participants in the D-process tasks and R-process tasks (part 1) and 26 (14 males) participants in the O-process tasks (part 2).

### Materials

The materials of the D-process and the R-process tasks consisted of Chinese characters for decomposition and reorganization both at the radical level and the stroke level (Luo et al., 2006; Wu et al., 2009). The materials of the O-process tasks consisted of the radical- or stroke-level separate components of Chinese characters utilized in the R-process tasks.

**Figure 1:**
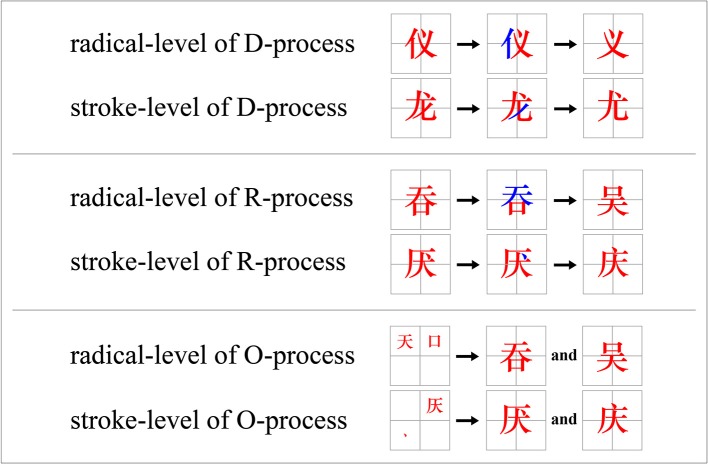
Examples of D-, R-, and O-process tasks. For the example of the radical level of the D-process, the initial state (task) was “仪” and the goal state (solution) was “义”; for the example of the stroke level of the D-process, the initial state was “龙” and the goal state was “尤”; for the radical level of the R-process, the initial state was “吞” and the goal state was “吴”; for the stroke level of the R-process, the initial state was “厌” and the goal state was “庆”; for the example of the radical level of the O-process, the initial state was “
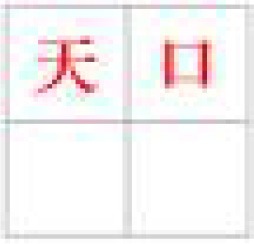
” and the goal state was “吞” and “吴” or “吴” and 吞”; for the example of the stroke level of the O-process, the initial state was “
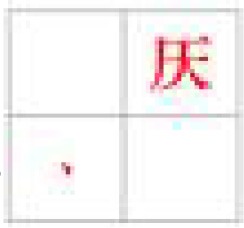
” and the goal state was “厌” and “庆” or “庆” and “厌.” The color was only for illustration, and that the characters appeared in black in the experiment.

The materials (Chinese characters) of D-process tasks of *radical-level* tasks can be divided in two sub-chunks (Figure 1). For example, the Chinese character “仪” (yi, meaning a person's appearance) can be decomposed into “义” (yi, meaning fairness) and “亻” (a radical, meaning a person). However, only “义” is an independent Chinese character, and the “亻” is a radical and not an independent character. This method of radical-level decomposition is the only solution; no other decomposition approaches could generate a correct solution. Thus, if we were required to generate a Chinese character by decomposing “仪,” we could generate the single result “义.” The *materials* of the D-process task of *stroke level* can be decomposed into several basic components. For example, the Chinese character “龙” (long, meaning dragon) can be decomposed into “尤” (you, meaning special) and “丿” (a stroke). Likewise, as the “丿” cannot be an independent character, the only correct and meaningful answer of generating a character by decomposing “龙” is “尤.” Utilizing other decomposition approaches with this character, whether at the radical level or stroke level, could not generate a meaningful and correct solution. Of particular importance is that whether the task was a radical-level or stroke-level task, the solution was exclusive and unambiguous.

For the *radical-level* condition of R-process tasks (see Figure 1), each character could be divided in two sub-chunks that could be reorganized into a new character. For example, the Chinese character “吞” (tun, meaning swallowing) could be divided into two parts: “天” (tian, meaning sky) and “口” (kou, meaning mouth). Then, putting the “口” above “天” could be organized in another way to generate a new character “吴” (wu, meaning a family name). The materials of R-process tasks of *stroke level* could thus be decomposed to two basic components and regrouped into a new meaningful character. For example, the character “厌” (yan, meaning disgusting) can only be decomposed to “
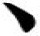
” and “厌” and then regrouped to generate the new character “庆” (qing, meaning celebrate). As with the D-process tasks, each material can generate only an unambiguous solution. Therefore, the R-process tasks consisted of two steps: chunk decomposition and reorganization. The decomposition step was similar to that of the D-process tasks, which required participants to break a character at the radical or stroke level. Unlike the task for the D-process, the task for the R-process did not require participants to decompose the character in such a way that one part was a meaningful character. The reorganization step was based on the results of the decomposition step, and it required participants to rejoin the dissociated parts to form a new character.

For the O-process tasks (see Figure 1), the same characters were utilized as for the R-process tasks; however, it exposed the two already separated components corresponding to either the radical- or the stroke-level decomposition. For instance, in contrast to the radical-level condition of the R-process tasks, wherein the Chinese character “吞” should be first decomposed into the parts “天” and “口” before the parts should be reorganized as the new character “吴,” in the O-process tasks, the two already decomposed components, “天” and “口,” were presented as the experimental materials in the *radical-level condition*. In the *stroke-level condition*, similar materials were used. For example, the “
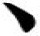
” and “厌” were directly presented as the experimental materials. Each task could generate only two meaningful and unambiguous solutions. The structure types of Chinese characters include the “left-and-right structure” and the “upper-and-lower structure.” If we provide the two components in a fixed space order and direction, such as from the left to right, the corresponding structure of the character (here, the “left-and-right structure”) would be easy to generate as the stimuli-presenting order and the method would provide some hint messages. For this reason, each of two components of the materials was pseudo-randomly put into one of the four boxes provided.

The familiarity of the Chinese characters was rated by three Chinese graduate students of psychology major on a 7-point scale (1 = extremely unfamiliarity, 7 = extremely familiarity). Chinese characters with neutral familiarity (*M* = 4.81, *SD* = 1.72, ranging from 3.61 to 6.32) and similar stroke numbers (*M* = 7.61, *SD* = 3.20, ranging from 4 to 11) were selected as experimental materials (see Supplementary Materials). There was no significant difference in the familiarity or stroke number of the different conditions. Finally, 20 Chinese characters (D-process task and R-process tasks) or materials (O-process tasks) were utilized in each condition. The total number of utilizing Chinese characters was 80 in the D- and R-process tasks and of materials was 40 in the O-process tasks.

### Procedure

Part 1: Both the D-process tasks and R-process tasks were within-participants designs, and the participants were required to perform both types of tasks. Prior to the formal tasks, there was a practice stage to make participants understand the tasks. During the experiment, the two types of tasks were presented in two separate blocks and the order of the two blocks was balanced across participants.

For each trial of the D-process tasks, a Chinese character was presented randomly in the center of the screen for 15,000 ms. Participants were asked to think of a meaningful character by removing a part of the character and pressing a response key with the right index finger as soon as possible when he/she generated an answer. In the instructions for participants, the difference between the radical- or stroke-level decomposition and organization was not explicitly mentioned or emphasized; participants were simply told that they could take away any parts they needed to in order to achieve the goal state. A block box was then presented immediately in the next picture, and the participant was required to write down the spelling of the solution in the box. Subsequently, the next trial would be presented and the participants were required to solve this task immediately. There were 40 trials in total. In the R-process tasks, a similar procedure was followed as for the D-process tasks. The only difference was that the participants were asked to think of another meaningful character by decomposing the target character in any necessary way first and then regrouping the components as another character without leaving any remnants. The reaction time (RT) and accuracy (ACC) were recorded by the E-prime procedure automatically.

**Figure 2:**
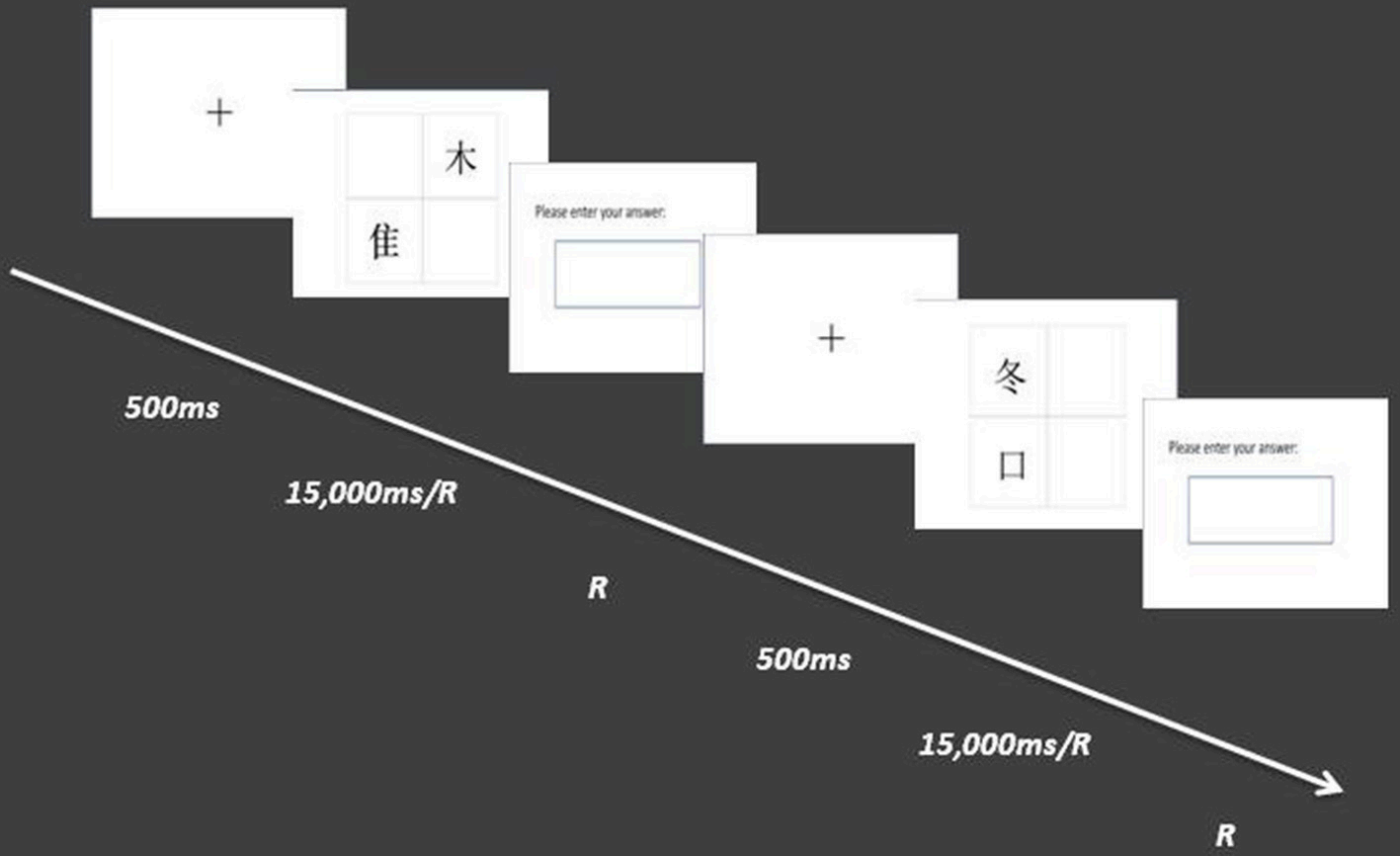
The procedure of experiment 3. the material was presented randomly in the center of the screen for 15,000 ms after the fixation. Participants were asked to think of two meaningful characters by combining the two displayed decomposed parts. By generating two meaningful characters through two different organizational methods, he/she should press a response key with the right index finger as soon as possible. A block box was then presented in the next picture immediately, and each participant was asked to write down the spelling of both characters in the box separated with a comma. The procedures of experiments 1 and 2 are similar with experiment 3.

Part 2: The task of O-process was a follow-up study based on the results of experiment 1 and 2 which have founded the different mechanisms between the D-process and R-process. Then, we wanted to know the purified mechanisms of the O-process separated out of R-process. The O-process tasks were within-participant designs. Prior to the formal tasks, there was a practice stage to enable participants to understand the tasks. In each trial of the O-process tasks, the material was presented randomly in the center of the screen for 15,000 ms. Participants were asked to think of two meaningful characters by combining the two displayed decomposed parts. By generating two meaningful characters through two different organizational methods, the participants were required to press a response key with the right index finger as soon as possible. A block box was then presented in the next picture immediately, and each participant was asked to write down the spelling of both characters in the box separated with a comma. The reaction time (RT) and accuracy (ACC) were recorded automatically by the E-prime software (see Figure 2).

## Results

First, we utilized the Kolmogorov–Smirnov (K-S) test to check if the data were distributed normally and the results indicated that all the data of accuracy and reaction time were distributed normally (*p*_all_ > 0.05).

**Figure 3:**
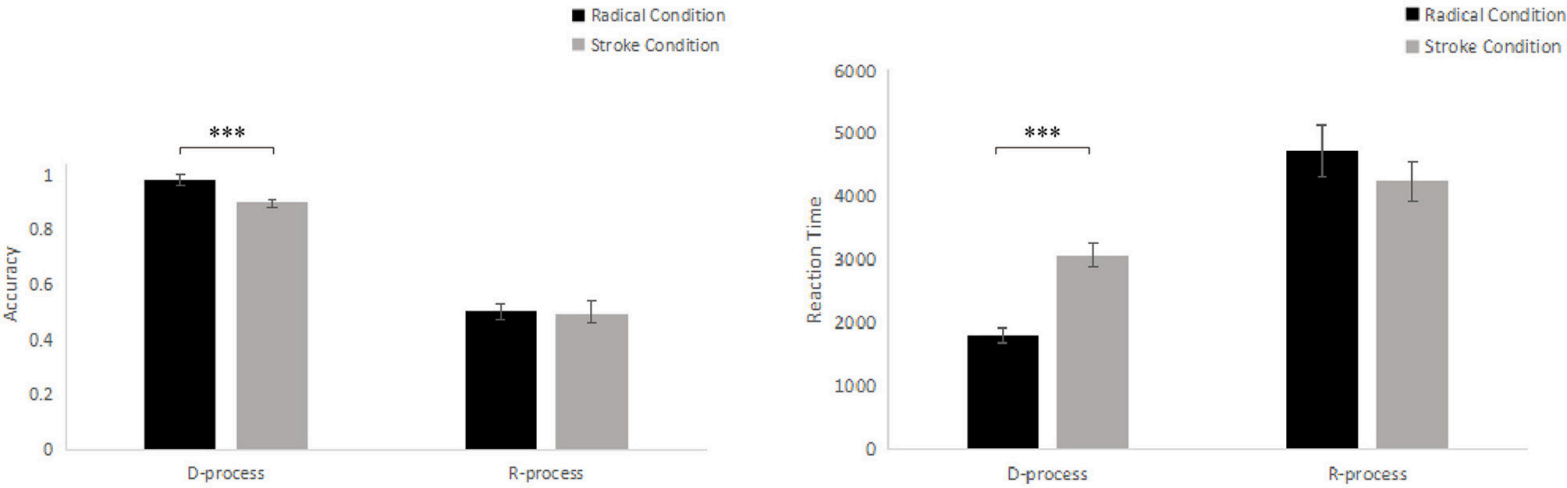
Comparisons of the accuracy and reaction time between the stroke level and the radical level of the D- and R-process tasks. The error bars (capper vertical bars) represent the standard error. ^***^ Indicates a significant difference at *p* < 0.001.

Part 1: In order to investigate if the tightness of CD altered the performance of D-process and R-process in different way, A 2 (tasks: D-process, R-process) × 2 (radical, stroke) repeated-measures ANOVA were conducted to examine the participants' accuracy (ACC) and the reaction time (RT) of the correct solutions. For accuracy (see Table 1 and Figure 3), the results showed significant main effects of tasks between D-process and R-process [*F*_(1, 24)_ = 205.23, *p* < 0.001, partial η2 = 0.90] and radical or stroke levels [*F*_(1, 24)_ = 4.93, *p* = 0.04, partial η2 = 0.17]. The interaction effect was marginally significant [*F*_(1, 24)_ = 3.39, *p* = 0.08, partial η2 = 0.12]. A simple main effect analysis showed significant differences between stroke level and radical level in the D-process (*P* < 0.001) and the accuracy in removing the radical condition (*M* = 98.40%, *SD* = 3.74%, *SEM* = 4.50%, *CI* = 96.86% ~ 99.94%) was significantly greater than those in removing the stroke condition (*M* = 90.00%, *SD* = 8.16%, *SEM* = 2.67%, *CI* = 86.63% ~ 93.37%); but did not show significant differences between stroke level (*M* = 49.20%, *SD* = 13.36%, *SEM* = 2.67%, *CI* = 43.69 ~ 54.71%) and radical level (*M* = 49.80%, *SD* = 22.52%, *SEM* = 4.50%, *CI* = 40.50 ~ 59.10%)in the R-process (*p* = 0.88).

**Table 1:** Reaction time and accuracy of the three tasks, with standard error in parentheses.

	**Radical condition (RT)**	**Stroke condition (RT)**	**Radical condition (ACC)**	**Stroke condition (ACC)**
D-process	1,797 ms (110 ms)	3,070 ms (179 ms)	98.40% (0.74%)	90.00% (1.63%)
R-process	4,730 ms (403 ms)	4,245 ms (312 ms)	49.80% (4.50%)	49.20% (2.67%)
O-process	6,405 ms (259 ms)	5,602 ms (274 ms)	62.20% (2.26%)	64.20% (2.41%)

For reaction time (see Table 1 and Figure 3), a 2 (tasks: D-process, R-process) × 2 (radical, stroke) repeated-measures ANOVA showed significant main effects of tasks between D- and R- process [*F*_(1, 24)_ = 45.77, *p* < 0.001, partial η2 = 0.66] and radical or stroke levels [*F*_(1, 24)_ = 7.28, *p* < 0.01, partial η2 = 0.23]. The interaction effect was significant [*F*_(1, 24)_ = 23.43, *p* < 0.001, partial η2 = 0.49]. A simple main effect analysis showed significant differences between stroke level and radical level in the D-process (*P* < 0.001) and the average reaction time of the correct solutions in removing the radical condition (*M* = 1,797 ms, *SD* = 548 ms, *SEM* = 110 ms, *CI* = 1,570 ms ~ 2,023 ms) was significantly shorter than in removing the stroke condition (*M* = 3,070 ms, *SD* = 897 ms, *SEM* = 179, *CI* = 2,699 ms ~ 3,440 ms); but did not show significant differences between stroke level (*M* = 4,245 ms, *SD* = 1,560 ms, *SEM* = 321 ms, *CI* = 3,600 ms ~ 4,889 ms) and radical level (*M* = 4,730 ms, *SD* = 2,015 ms, *SEM* = 403 ms, *CI* = 3,898 ms ~ 5,561 ms) in the R-process (*p* = 0.11).

**Figure 4:**
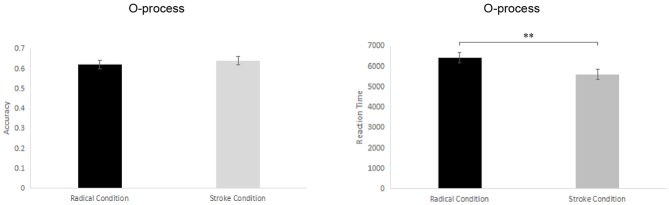
Comparisons of the accuracy and reaction time between the stroke level and the radical level of the O-process tasks. The error bars (capper vertical bars) represent the standard errors. ^**^ Indicates a significant difference at *p* < 0.01.

Part 2: For the O-process tasks paired-sample *t*-tests were conducted to examine the participants' accuracy (ACC) and the reaction time (RT) of the correct solutions between the stroke level and the radical level. The results (see Table 1 and Figure 4) showed no significant difference in the accuracy in the organization radical condition (*M* = 62.20%, *SD* = 11.49%, *SEM* = 2.25%, *CI* = 57.56 ~ 66.84%) and the stroke condition (*M* = 64.20%, *SD* = 12.30%, *SEM* = 2.41%, *CI* = 59.23 ~ 69.17%), *t*_(25)_ = 0.79, *p* = 0.44, *Cohen d* = 0.17. However, there was a significant difference in the reaction time of the correct solutions between the organization radical condition (*M* = 6,405 ms, *SD* = 1,458 ms, *SEM* = 259 ms, *CI* = 5,816 ms ~ 6,994 ms) and the stroke condition (*M* = 5,602, *SD* = 1,397 ms, *SEM* = 274, *CI* = 5,038 ~ 6,167 ms), *t*_(25)_ = 3.52, *p* = 0.002, *Cohen d* = 0.56. The average reaction times of the correct solutions in the organization stroke condition were significantly shorter than in the organization radical condition. The reaction times were thus more sensitive indicators than the accuracy response to these tasks.

## Discussion

The present study replicated previous findings, demonstrating that when the to-be-decomposed part was an element/stroke, the problem was more difficult to solve than when the to-be-removed element was a chunk/radical in the D-process task (Luo et al., 2006). However, when the task was to regroup the elements of the target character as a new character (as in the R-process tasks), the level of chunk tightness (i.e., the radical- or the stroke-level chunk decomposition and reorganization) made no difference in the accuracy and the reaction time. Moreover, the stroke-level organization of elements into meaningful chunks/characters was found to require a shorter reaction time than the radical-level organization in the O-process tasks, which focused on a relatively pure process of organization, implying that the stroke-level organization could even be easier than the radical-level organization. These results demonstrated that the level of chunk tightness in decomposition altered the performance of the D-process and the R-process in different ways, showing that the D-process and the R-process could be distinctive processes.

As in the present study, Knoblich and colleagues' original experiment (Knoblich et al., 1999) also involved the processes of chunk decomposition and reorganization. However, unlike the R-process task in the present study, which revealed that the chunk/radical level and the component/stoke level chunk decomposition and reorganization made no difference in participants' problem-solving performance, Knoblich and colleagues' study did find that it was more difficult for subjects to transform “**XI** = **III** + **III**” into “**VI** = **III** + **III**” than to transform “**IV** = **III** + **III**” to “**VI** = **III** + **III**,” which implied that the factor of chunk tightness could also affect the performance of chunk decomposition and reorganization. One possible reason for this inconsistency was the reorganization process of the control condition in Knoblich and colleagues' study (i.e., to reorganize **IV** as **VI**) was so obvious and easy that it failed to provide a reasonable reference condition for examining the effects of reorganization. In contrast, the reorganization task using Chinese characters contained so many different items, variations and possibilities that the subjects could not determine a direct solution to the problem, instead having to search the possible solutions in the problem space. In this sense, the chunk decomposition and reorganization task using Chinese characters as materials could be considered to be more suitable to simulate and represent the problem-solving process than the matchstick arithmetic task.

An unanswered question regarding the R-process task is the following. This task involves both the process of decomposition and that of reorganization. From the results for the D-process, we already know that the stroke-level decomposition was significantly more difficult than the radical-level decomposition. Why, then, was this stroke vs. radical difference not found in participants' performance in the R-process task? Although the radical-level decomposition was easier than the stroke-level decomposition, this advantage could be concealed or at least offset by the subsequent reorganization process. The results on the O-process that involved only a chunk of the organization process indicated that, for the RTs, it might be easier for an individual to realize stroke-level organization than radical-level organization. This finding implied that the effects of radical- and stroke-level manipulations in CD and in chunk reorganization could be reversed. As the radical was a meaningful chunk it was easier to decomposing for radical level and as the same reason it was more difficult to regroup other new meaningful chunk. It may be this effect that eventually overrode the superior performance of radical-level manipulation over stroke-level manipulation in the R-process task.

The underlying reason why the stroke-level manipulation is not more difficult or is even easier than the radical-level manipulation could be related to the global-first principle of perception. According to this principle, the perceptual response to a global shape is more preferential than to a local shape, and the influence of the global shape on the local shape is stronger than the reverse influence. In addition to Navon (1977)'s original research that found participants were more rapid and accurate in identifying the large letter than the small ones that formed the large letter, recent study revealed local details encoded in lower-order visual areas are unconsciously processed before being automatically and rapidly combined into global information in higher-order visual areas, where conscious percepts emerge (Campana et al., 2016). From this global-first point of view, the influence, or more specifically, the constraining effect, of the radicals or sub-chunks on R- process could be even more robust than that of strokes or other more basic chunk elements because the global effects of the former could be more dominant than those of the latter.

In summary, this study was the first to reconsider the concept of CD proposed by Knoblich et al. (1999, 2001) and to assess the theory behind it. Our results demonstrated that the tightness of CD altered the performance of D-process tasks and R-process tasks in different ways perhaps as the reason that the R-process as a holistic regrouping process may follow a global-first principle rather than one based on chunk tightness. Thus, this study suggested that the D- and R-processes may jointly contribute to this type of insight problem solving. New concepts such as “chunk restructuring” may be more suitable to account for these processes which at least contain two distinctive aspects or processes: the decomposition aspect or process (the D-process) and the reorganization aspect or process (the R-process).

## Author contributions

XW and JL designed the experiment. JL and JX supervised the research procedure. XW, MH, and YZ collected and analyzed the data. XW, JL, and JX wrote the manuscript.

### Conflict of interest statement

The authors declare that the research was conducted in the absence of any commercial or financial relationships that could be construed as a potential conflict of interest.
