# Supplementary material for: Decomposing a Chunk into Its Elements and Reorganizing Them As a New Chunk: The Two Different Sub-processes Underlying Insightful Chunk Decomposition
Source: Front Psychol. 2017 Nov 14;8:2001. doi: 10.3389/fpsyg.2017.02001 (PMC5694466; doi:10.3389/fpsyg.2017.02001)
Supplement: Supplementary file 1 [file Presentation1.PDF]

Part of materials：

Remove radical materials：

唯，售　　囹，呤　　固，咕　　部，陪

吞，吴　　含，吟　　机，朵　　集，椎

员，呗　　叨，召　　只，叭　　垦，垠

Remove stroke materials：

戎，戌　　庄，压　　主，玉　　百，自

汗，江　　环，坯　　庆，厌　　半，平

巫，坐　　同，回　　杠，杆　　泠，冷

Decompose radical materials：

打，丁　　记，己　　仪，义　　刊，干

阡，千　　冯，马　　郑，关　　掉，卓

依，衣　　阳，日　　都，者　　请，青

Decompose stroke materials：

丢，去　　舟，丹　　并，开　　再，冉

吏，史　　龙，尤　　币，巾　　衰，哀

用，月　　习，刁　　亚，业　　友，又
