# Supplementary material for: Decomposing a Chunk into Its Elements and Reorganizing Them As a New Chunk: The Two Different Sub-processes Underlying Insightful Chunk Decomposition
Source: Front Psychol. 2017 Nov 14;8:2001. doi: 10.3389/fpsyg.2017.02001 (PMC5694466; doi:10.3389/fpsyg.2017.02001)
Supplement: Supplementary file 2 [file DataSheet1.pdf]

Rem R(rt)	Rem S(rt)	Dec R (rt)	Dec S(rt)	RRA	RSA	DRA	DSA
7127.0000	3554.5455	996.0526	2784.9474	0.30	0.55	1.00	0.95
5322.4000	4340.8571	2164.6471	4189.0588	0.50	0.35	0.85	0.85
4028.0000	4944.5833	1517.0526	3389.5263	0.75	0.60	0.95	0.95
4652.6667	6213.7273	2608.5500	3965.8947	0.30	0.55	1.00	0.95
1274.7500	2855.8571	1855.6000	2604.2222	0.20	0.35	1.00	0.90
5166.2000	4163.5556	2893.5000	3778.4667	0.25	0.45	1.00	0.75
7369.1667	4175.5000	2595.8000	3961.7222	0.30	0.40	1.00	0.90
6987.7778	5251.5000	1674.7500	3836.2500	0.45	0.40	1.00	0.80
6042.0769	5137.1818	2199.8000	3149.5294	0.65	0.55	1.00	0.85
5310.8000	5318.8333	1917.9000	3075.2000	0.25	0.65	1.00	1.00
5825.1538	6420.0000	1390.1500	1395.2632	0.65	0.50	1.00	0.95
7461.0000	6121.5000	1765.2778	2349.0000	0.20	0.30	0.90	0.90
5169.2143	2673.9167	2577.5500	3730.1875	0.70	0.60	1.00	0.80
4871.1765	3609.2500	1439.3000	2955.3889	0.85	0.60	1.00	0.90
2192.0588	2523.0625	1189.4500	2232.9000	0.85	0.80	1.00	1.00
3844.2353	5485.0000	969.3500	2465.3500	0.85	0.50	1.00	1.00
5837.1111	4598.5000	1202.4000	2963.8421	0.45	0.30	1.00	0.95
3993.0000	6098.0000	1828.7000	2454.5500	0.40	0.55	1.00	1.00
5520.2000	5056.7500	1526.7500	3956.8824	0.25	0.40	1.00	0.85
5603.2222	4736.0000	1567.6000	3378.6429	0.45	0.50	1.00	0.70
730.6875	929.4545	1656.4500	1266.5263	0.80	0.55	1.00	0.95
7318.5000	5370.8889	2642.4737	4048.6250	0.70	0.45	0.95	0.80
1096.3636	1118.6154	1154.3000	1227.8889	0.55	0.65	1.00	0.90
1862.6364	1741.1818	2007.8421	3470.7895	0.55	0.55	0.95	0.95
3637.0000	3683.0000	1581.8000	4118.5263	0.25	0.20	1.00	0.95

Org R(rt)	Org S(rt)	ORA	OSA
5835.57	3469.60	0.75	0.55
5713.47	4361.13	0.75	0.75
3827.88	4614.28	0.50	0.70
5193.10	4670.16	0.55	0.65
4571.10	4263.22	0.55	0.75
5330.53	4666.78	0.55	0.60
4945.65	4916.00	0.65	0.75
3855.26	2529.00	0.75	0.70
6306.64	5143.88	0.60	0.40
9395.71	7545.10	0.75	0.85
5273.13	5080.00	0.70	0.45
6615.88	6565.79	0.50	0.45
7635.54	6307.00	0.65	0.60
7885.20	6109.90	0.35	0.55
8115.29	8512.08	0.80	0.85
7417.43	6607.89	0.40	0.45
7132.83	6327.36	0.60	0.75
6168.53	5851.87	0.70	0.70
6638.23	6295.64	0.50	0.65
8372.92	5380.80	0.75	0.60
7668.91	7681.75	0.70	0.75
5092.27	4957.13	0.65	0.65
8269.00	6186.93	0.55	0.75
7356.80	4838.29	0.60	0.50
6475.33	4834.80	0.70	0.65
5441.60	7943.50	0.62	0.64

DATA:

Rem R(rt) =removing radical RT

Rem S(rt) =removing stroke RT

Dec R (rt)=decomposing radical RT

Dec S (rt)=decomposing stroke RT

RRA =removing radical ACC

RSA =removing stroke ACC

DRA=decomposing radical ACC

DSA=decomposing stroke ACC

Org R(rt) = organizing radical RT

Org S(rt)=organizing stroke RT

ORA =organizing radical ACC

OSA =organizing stroke ACC
